# Facial Neuralgia Consequent upon Pregnancy

**Published:** 1889-10

**Authors:** W. W. Allport

**Affiliations:** Chicago


### COMMUNICATIONS.

### Facial Neuralgia Consequent upon Pregnancy.

BY W. W. ALLPORT, M.D., D.D.S., OF CHICAGO.

Read before the Section of Dental and Oral Surgery at the fortieth annual meeting of the American Medical Association, at Newport, June, 1889.

In the paper that I am about to present to this section I propose, very briefly, to discuss the nature and causes of facial neuralgia consequent upon pregnancy.

For the term neuralgia, I have never seen a definition that exactly suited me, nor am I satisfied with any definition that I myself can make. But in a general way it may be said that neuralgia is an acute intermittent pain, carried in irregular and often in divergent currents through the nerves and their branches, the result of an unnatural disturbance of molecular vital forces; or, it may be said to be a nerve's expression of some pathological condition which may be contiguous to, or far removed from, the point of expression. Pain is a nerve's expression of disease as words are expressions of thought. It is a symptom, or an informer of disease, imploring help.

The causes of neuralgia are manifold. Among the most prominent may be mentioned sudden changes of temperature, pressure upon any portion of a nerve trunk or its branches. This may be from a local deposit or growth of any kind; or from arterial tension by an increased volume of blood ; or it may be produced by an opposite, an anæmic condition, a deficiency of blood ; an impoverished condition of blood, indigestion ; or, in fact, any thing that produces such a disproportion of the standard constituents of the blood as will disturb nutrition, or by an impairment or decay of tissues. Upon this latter point Anstie says: "Amongst the neuralgias that are the most *absolutely* agonizing are those which occur under circumstances of impaired nutrition incident to bodily decay," and especially is this so when it occurs at parts at the peripheral end of the nerve.

These are by no means all of the causes of neuralgia, but I have named enough for the object I have in view, namely, to show that facial neuralgia due to pregnancy, is not due as is generally supposed, from reflex pain caused by disturbances in the uterus, or from pressure upon nerve trunks in its immediate locality by the increased weight of this organ during the period of gestation.

It is a fact, I believe, that women while in this condition suffer more from neuralgia in the upper than in the lower extremity. If this be so there must be a reason for it, and it can hardly be from *weight* pressure of the uterus, for were this the cause of neuralgic pain we should naturally look for its prevalence in the lower rather than the upper extremities, while in fact, it is in the face, cranium, and teeth that it is most frequently manifest. Then, too, it is a long way and a very circuitous route from the uterus to the branches of the fifth pair of nerves in the face and its surroundings, and it is difficult to see why these, rather than other nerves of the body should be most frequently selected for reflex pain under uterine irritation or pressure.

Although there are exceptions to the rule, I think it is an admitted fact, that while there is usually an increased volume of blood in women in this condition, the increase is in its white rather than its red corpuscles, its life-giving properties. At the very period of a woman's life when it would seem to be most important that the proportion of life-giving properties of her blood should be the richest it generally seems to be the poorest. When her system is called upon to sustain, in addition to her own, a new life, her blood is deficient in the life-giving properties to properly nourish her own body, to say nothing of the child she is to bear. The superabundance of serum in her blood may make her plump and full, yet she is usually pale, evincing a lack of vitality, or proper tissue nourishment.

Another important fact bearing on the point in question is that all below the diaphragm is in a constant state of venous hyperæmia, while that above is in a constant state of arterial hyperæmia, and arterial tension with its muscular expansion and contraction is always a fruitful source of nerve irritation and pain. This is doubly true when the arterial hyperæmia occurs in organs like the mouth, where among the teeth there is almost always some pathological condition present.

Then, too, it is a well-known fact that during the period of gravidity most women are troubled with irritability of the nerves of the mucous membrane of the stomach ; so much so that at times it is difficult for them to retain a sufficient amount of food for proper nourishment. It is much easier, as well as more rational, to conclude that facial neuralgia in pregnancy is reflected from the nerve irritation of the stomach rather than from the uterus, for irritation of the stomach, or indigestion, is a well-known cause of this symptom. Neuralgia in the lower part of the abdomen, the inguinal regions, etc., is not uncommon in non-pregnant women. In pregnant women, however, these neuralgias are said to be seldom seen. If the neuralgia of pregnancy were a uterine reflex, its *locale* would naturally seem to be near to the uterus rather than distant from it, as in the prevalent facial neuralgia of this condition. In fact, the entire upper portion of the alimentary canal, including the mouth, is usually in an irritable condition, while the lower part is apt to be in a sluggish and torpid condition.

In a majority of cases the sweat glands of pregnant women are found to be in an abnormal state of functional activity ; of course the face does not escape this condition, and no one need be told that with its almost constant exposure to atmospheric influences, the nerves of the face are particularly liable to those atmospheric impressions that are always productive of those molecular changes peculiar, or essential, to nerve pain.

Gingivitis is another source of irritation to which these subjects are peculiarly liable, the majority not escaping it. Such is the nervous and vascular connection of the gums with the pericemental membrane that the disease usually extends to the latter organ, and it not infrequently happens that the entire denture becomes not only loose, but the pressure on the teeth produces acute pain consequent upon severe inflammation in the pericemental membrane. This membrane in every way presents the hyperæmic condition that we would expect to be productive of reflex nerve pain. Besides this the swollen condition of the membranes surrounding the apical foramen of the tooth so strangles the vascular and nerve supply of the tooth as to seriously interfere with their functions, and must therefore produce irritation at the nerve peripheries. Consequent upon this, as well as upon other causes, proper nourishment of the tooth structure is cut off and the tooth is not only rendered more liable to irritation and decay from the action of external agents, but such starved condition and retrograde metamorphosis of the nerve fibrils of the tooth suructure is established as to be prolific of the agonizing neuralgia spoken of by Anstie which occurs under circumstances of impaired nutrition incident to wasting of tissue or bodily decay.

